# Clinical comparisons between previously diagnosed SLE and newly diagnosed SLE by kidney biopsy

**DOI:** 10.1186/s13317-020-00140-2

**Published:** 2020-12-02

**Authors:** Pantipa Tonsawan, Kittisak Sawanyawisuth

**Affiliations:** Department of Medicine, Faculty of Medicine, Khon Kaen University, Khon Kaen, 40002 Thailand

**Keywords:** Hypertension, Systemic lupus erythematosus, Diagnosis

## Abstract

**Background:**

Lupus nephritis is a type of major organ involvement in systemic lupus erythematosus (SLE) patients that leads to higher rates of morbidity and mortality and may present initially in 28% of SLE patients. However, there are limited data available on clinical differences or predictors for biopsy-proven lupus nephritis in established versus newly diagnosed SLE cases.

**Methods:**

Adult patients undergoing kidney biopsy for the first time with a diagnosis of lupus nephritis were eligible for inclusion. Patients were categorized into two groups: those with previously diagnosed SLE and those with newly diagnosed SLE by kidney biopsy. Factors associated with newly diagnosed SLE were determined using logistic regression analysis.

**Results:**

There were 68 patients diagnosed with lupus nephritis by kidney biopsy. Of those, 31 cases (45.58%) were newly diagnosed. The newly diagnosed SLE group was significantly older (36.87 vs 30.95 years) and had a lower proportion of females (74.19% vs 91.89%) than the previously diagnosed group. A new-onset hypertension was the only factor independently associated with newly diagnosed SLE by kidney biopsy. The adjusted odds ratio (95% CI) was 5.152 (1.046, 25.363).

**Conclusions:**

Nearly half of the biopsy-proven lupus nephritis cases in this study were patients with newly diagnosed SLE. Patients with previously diagnosed SLE and newly diagnosed SLE by kidney biopsy had clinical differences.

## Introduction

Systemic lupus erythematosus (SLE) is an autoimmune disease that may involve several organs. Renal involvement or lupus nephritis affects approximately 50% of SLE patients [1] and is significantly related with mortality (adjusted hazard ratio of 1.65 with 95% CI of 1.03, 2.66) [2]. Additionally, end-stage renal disease may develop in 10% of SLE patients with renal involvement [3].

 Lupus nephritis has varying clinical presentations and outcomes, ranging from asymptomatic urine abnormalities to rapid decline in kidney function. Clinical renal parameters for active lupus nephritis in established SLE patients can be evaluated by urinalysis and serum creatinine measurement [1]. Previous studies found that the rate of lupus nephritis increased from 16 to 28% in 10 years and it increased from 32 to 47% in nine years [4–6]. Evidence of proteinuria with or without active urine sediments/cellular casts or unexplained serum creatinine values may indicate lupus nephritis in patients with previously diagnosed SLE. However, kidney biopsy remains the gold standard for diagnosis [1]. Twenty-eight percent of young patients with SLE may present with lupus nephritis initially. However, data are limited with regard to clinical differences between patients with renal involvement who have previously been diagnosed with SLE versus those who are newly diagnosed [5]. This study aimed to (1) evaluate clinical differences between both groups and (2) identify any clinical factors that are predictive of newly diagnosed SLE by lupus nephritis.

## Methods

This was a subgroup analysis of a previous study published in 2019 [7]. We included adult patients who underwent kidney biopsy for the first time with a diagnosis of lupus nephritis classified by a renal pathologist according to the 2003 International Society of Nephrology/Renal Pathology Society (ISN/RPS) classification system (n = 68) [8]. We excluded those with diagnoses other than lupus nephritis (n = 137). The eligible patients were categorized into two groups: those with previously diagnosed SLE and those with newly diagnosed SLE by the kidney biopsy.

Clinical factors were evaluated including baseline characteristics, co-morbid diseases, new-onset hypertension, physical signs, and laboratory results. New-onset hypertension was defined as blood pressure 140/90 mmHg or over without other causes of hypertension. Laboratory results included serum creatinine, glomerular filtration rate, 24-h proteinuria, serum albumin, serum cholesterol, HBV/HCV/HIV infection, urinalysis results, and pathological results of kidney biopsy.

### Statistical analysis

Descriptive statistics were used to compare differences between patients with previously diagnosed and newly diagnosed SLE. Factors associated with newly diagnosed SLE were identified using logistic regression analysis. Factors with a p value of less than 0.20 by univariate logistic regression analysis were included in subsequent multivariate logistic regression analysis. A stepwise method was used to determine the remaining factors for newly diagnosed SLE. The final model was tested for goodness of fit using the Hosmer–Lemeshow method. Data are presented as mean (SD), number (proportion), and unadjusted/adjusted odds ratio with 95 confidence interval (CI). Statistical analyses were performed using STATA version 10.1 (College Station, Texas, USA).

## Results

There were a total of 68 patients diagnosed with lupus nephritis by kidney biopsy, 31 (45.58%) of whom were newly diagnosed of SLE by renal pathology. Class IV lupus nephritis was found in most patients in both groups, but there was a higher proportion in the group that had previously been diagnosed (70.27% vs 58.06%; p 0.401). With regard to clinical features, the two groups differed significantly in terms of age and sex by descriptive statistics (Table 1). The newly diagnosed group was significantly older (36.87 vs 30.95 years) and had a lower proportion of females (74.19% vs 91.89%) than the previously diagnosed group (Table 1).

**Table 1:** Clinical features of systemic lupus erythematosus (SLE) patients who underwent kidney biopsy categorized by newly diagnosed SLE and previously diagnosed SLE

Factors	Previously diagnosed SLE n = 37	Newly diagnosed SLE n = 31	p value
Age, years	30.95 (12.38)	36.87 (12.32)	0.046
Female sex, n (%)	34 (91.89)	23 (74.19)	0.048
Body mass index, kg/m^2^	25.00 (6.12)	26.37 (4.75)	0.796
Diabetes mellitus, n (%)	1 (2.70)	0	0.999
Hypertension, n (%)	23 (62.16)	16 (51.61)	0.381
New-onset hypertension, n (%)	3 (8.11)	7 (22.58)	0.167
Stroke, n (%)	0	1 (3.23)	0.456
Systolic blood pressure, mmHg	134.35 (19.93)	134.22 (21.65)	0.980
Diastolic blood pressure, mmHg	83.48 (15.89)	84.38 (14.74)	0.810
Serum creatinine, mg/dL	1.51 (1.70)	1.82 (2.72)	0.838
Glomerular filtration rate, mL/min/1.73m^2^	79.48 (41.16)	78.51 (44.18)	0.925
24-h proteinuria, g/day	3.55 (2.21)	5.09 (3.78)	0.054
Serum albumin, g/dL	2.51 (0.87)	2.58 (0.62)	0.732
Serum cholesterol, mg/dL	314.56 (110.83)	354.58 (122.97)	0.083
HBV infection, n (%)	1 (2.70)	1 (3.23)	0.999
Crescent, n (%)	11 (29.73)	7 (22.58)	0.506
RBC in urine > 50 cells/hpf, n (%)	4 (10.81)	6 (19.35)	0.494
Pathological types, n (%)			0.401
Class II	0	1 (3.23)	
Class III	1 (2.70)	2 (6.45)	
Class IV	26 (70.27)	18 (58.06)	
Class IV plus V	0	2 (6.45)	
Class V	10 (27.03)	8 (25.81)	

Data presented as mean (SD) unless indicated otherwise

There were five factors with p values less than 0.20 by univariate logistic regression analysis: age (0.058), sex (0.060), new-onset hypertension (0.106), 24-h proteinuria (0.066), and serum cholesterol (0.168). These factors were subjected to stepwise multivariate logistic regression analysis, after which three remained in the final model: age, new-onset hypertension, and 24-h proteinuria (Table 2). Only new-onset hypertension was independently associated with newly diagnosed SLE by kidney biopsy. The adjusted odds ratio (95% CI) was 5.152 (1.046, 25.363) with a p value of 0.044. The Hosmer–Lemeshow Chi square of the final model was 4.22 (p 0.836).

**Table 2:** factors remaining after stepwise logistic regression analysis for newly diagnosed systemic lupus erythematosus by kidney biopsy

Factors	Unadjusted odds ratio (95% confidence interval)	Adjusted odds ratio (95% confidence interval)
Age	1.040 (0.998, 1.083)	1.041 (0.991, 1.093)
24-h proteinuria	1.196 (0.987, 1.450)	1.142 (0.939, 1.388)
New-onset hypertension	3.305 (0.775, 14.091)	5.152 (1.046, 25.363)

## Discussion

This study found that lupus nephritis may first present as newly diagnosed SLE in 45.58% of cases. Note that the rate here was drawn from patients who underwent kidney biopsy. This proportion was higher than has been previously reported [5, 9]. Previous studies have found lupus nephritis as a first presentation in 28–31% of childhood-onset and 3% of older-onset SLE patients. These differences may be due to differences in age and indications for kidney biopsy. In our study, patients were in their third decade of life and had definite diagnoses of lupus nephritis by kidney biopsy. Differences in ethnicity may be another explanation [10, 11].

There were several factors that could potentially differ in patients previously diagnosed with SLE and those who were newly diagnosed by kidney biopsy including age, sex, 24-h proteinuria, and serum cholesterol (Table 1). There were two significant factors between both groups: age (p 0.046) and sex (p 0.048). The newly diagnosed SLE by lupus nephritis had older age but lower proportion of female sex than the previously diagnosed with SLE. These findings may be related to sex differences. The newly diagnosed SLE group comprised of more men than the previously diagnosed SLE group (25.81% vs 8.11% as shown in Table 1). Previous studies showed that men patients with SLE were older (40 vs 36 years; p 0.006) and had more renal involvement (54.2% vs 29.9%; p < 0.0001) than female patients [12, 13].

Even though age and sex were significantly different between both studied groups, only new-onset hypertension was an independent factor for newly diagnosed SLE or first-time presentation with lupus nephritis after adjusted for other factors by multivariate logistic regression analysis (Table 2). Though the new-onset hypertension may be an indicator for renal involvement in previously diagnosed SLE patients, this study also found that it may be an indicator for newly diagnosed SLE as well. A previous study found that patients with hypertension were 3.39 times more likely to have proliferative lupus nephritis than those without [14]. In this study, newly diagnosed lupus nephritis was proliferative in approximately 70% of cases, indicating that new-onset hypertension may be a strong predictor for proliferative lupus nephritis. It is well established that delays in active lupus nephritis treatment leads to deterioration of kidney function and poor outcomes [15]. Hence, prompt initiation of proper immunosuppressive therapy should be emphasized, particular in patients who present with new-onset hypertension, urine abnormalities, and serologic markers of lupus.

Another study found that persistent hypertension was related to longer disease duration with a coefficient of 0.06 (p 0.04) [16]. As in this study, persistent hypertension was more prominent in patients with previously diagnosed SLE with lupus nephritis than in those newly diagnosed (62.16% vs 51.61%). Note that several diagnostic features for lupus nephritis did not differ between the two groups including crescent, serum albumin, and hematuria (Table 1). We also entered these factors into the stepwise model and found no statistical significance (data not shown).

The main strength of this study was that diagnosis of lupus nephritis was made based on kidney biopsy. However, it was limited in that the predictive model was calculated using only clinical criteria, as we lacked the data to analyze other variables, such as genetic factors, that may be related to lupus nephritis [17]. However, the results of this study may apply in resource-limited settings that mainly rely on clinical evaluation. Finally, there were few patients with new-onset hypertension resulting slightly wide 95% CI but it was statistically significant (p 0.044). Further studies may also be required to confirm the results of this study.

Nearly half of the biopsy-proven lupus nephritis cases in this study were patients with newly diagnosed SLE. Patients with previously diagnosed SLE and newly diagnosed SLE by kidney biopsy had clinical differences.

## Data Availability

Data were available upon request.

## References

[1] Almaani S, Meara A, Rovin BH (2017). Update on lupus nephritis. Clin J Am Soc Nephrol.

[2] Danila MI, Pons-Estel GJ, Zhang J, Vila LM, Reveille JD, Alarcon GS (2009). Renal damage is the most important predictor of mortality within the damage index: data from LUMINA LXIV, a multiethnic US cohort. Rheumatology (Oxford).

[3] Alarcon GS (2011). Multiethnic lupus cohorts: what have they taught us?. Reumatol Clin.

[4] Kasitanon N, Magder LS, Petri M (2006). Predictors of survival in systemic lupus erythematosus. Medicine (Baltimore).

[5] Cervera R, Khamashta MA, Font J (2003). Morbidity and mortality in systemic lupus erythematosus during a 10-year period: a comparison of early and late manifestations in a cohort of 1,000 patients. Medicine (Baltimore).

[6] To CH, Petri M (2005). Is antibody clustering predictive of clinical subsets and damage in systemic lupus erythematosus?. Arthritis Rheum.

[7] Tonsawan P, Puapairoj A, Chan-On C (2019). ANA should be performed in patients without previous history of SLE who underwent kidney biopsy. Nephro-Uro Mon.

[8] Weening JJ, D'Agati VD, Schwartz MM (2004). The classification of glomerulonephritis in systemic lupus erythematosus revisited. J Am Soc Nephrol.

[9] Mok CC, Tang SS (2004). Incidence and predictors of renal disease in Chinese patients with systemic lupus erythematosus. Am J Med.

[10] Wang F, Wang CL, Tan CT, Manivasagar M (1997). Systemic lupus erythematosus in Malaysia: a study of 539 patients and comparison of prevalence and disease expression in different racial and gender groups. Lupus.

[11] Alarcon GS, McGwin G, Petri M (2002). Baseline characteristics of a multiethnic lupus cohort: PROFILE. Lupus.

[12] Ramírez Sepúlveda JI, Bolin K, Mofors J (2019). Sex differences in clinical presentation of systemic lupus erythematosus. Biol Sex Differ.

[13] Tan TC, Fang H, Magder LS, Petri MA (2012). Differences between male and female systemic lupus erythematosus in a multiethnic population. J Rheumatol.

[14] Lydia A, Saraswati MH, Dharmeizar D, Saraswati M, Setiati S (2018). Diagnostic determinants of proliferative lupus nephritis based on clinical and laboratory parameters: a diagnostic study. Acta Med Indones.

[15] Korbet SM, Lewis EJ, Schwartz MM, Reichlin M, Evans J, Rohde RD (2000). Factors predictive of outcome in severe lupus nephritis. Lupus Nephritis Collaborative Study Group. Am J Kidney Dis.

[16] Shaharir SS, Mustafar R, Mohd R, Mohd Said MS, Gafor HA (2015). Persistent hypertension in lupus nephritis and the associated risk factors. Clin Rheumatol.

[17] Aulakh R, Singh S (2008). Strategies for minimizing corticosteroid toxicity: a review. Indian J Pediatr.

